# The Telecardiology Revolution: From Emergency Management to Daily Clinical Practice

**DOI:** 10.3390/jcm11071920

**Published:** 2022-03-30

**Authors:** Silvana De Bonis, Nadia Salerno, Antonio Bisignani, Antonella Verta, Cristina Capristo, Antonio Capristo, Gennaro Sosto, Sabato Sorrentino, Giovanni Bisignani

**Affiliations:** 1 Department of Cardiology, “Ferrari” Hospital, Castrovillari, 87012 Cosenza, Italy; silvanadebonis68@gmail.com; 2 Division of Cardiology, Department of Medical and Surgical Sciences, “Magna Graecia” University, 88100 Catanzaro, Italy; nadia.salerno@unicz.it (N.S.); sabatosorrentino@hotmail.com (S.S.); 3 Department of Cardiovascular and Thoracic Sciences, Institute of Cardiology, Catholic University of the Sacred Heart, Largo Agostino Gemelli, 00168 Rome, Italy; abisignani@hotmail.it; 4 Clinical Engineering, Azienda Sanitaria Provinciale (ASP) di Cosenza, 87100 Cosenza, Italy; antonella_verta@yahoo.it (A.V.); cristinacapristo@libero.it (C.C.); antoniocapristo@gmail.com (A.C.); 5 Azienda Sanitaria Locale (ASL) Napoli 3, 80045 Pompei, Italy; gennarososto@tiscali.it

**Keywords:** telecardiology, telemedicine, electrocardiogram, acute coronary syndrome

## Abstract

Aims: Telecardiology is one of the most widespread applications of telemedicine. We aimed to report the design and development of a telecardiology system in the sanitary district of Cosenza, one of the largest in Italy, with a complex orography, and healthcare reorganization needs, for the management of the emergency network and daily clinical practice. Methods: Our telecardiology network connects 8 hospitals, 9 first aid centers, 20 local 118-EMS stations, 1 helicopter station, 8 hospital emergency departments, 59 hospital departments, and 3 catheterization laboratories. All data are centralized on a dedicated server, accessible from any location for real-time assessment. The quality, source, and timing of the electrocardiograms transmitted were evaluated. Results: From October 2015 to December 2019, a total of 389,970 ECGs were transmitted. The quality of ECGs was optimal in 52%, acceptable in 42%, and poor in 6% of the cases. The number of poor-quality ECGs was only 3% in the last 2 years. Out of the total, 145,097 (37.2%) were transmitted from the emergency departments and 5318 (1.4%) from the 118-EMS. Of interest, a sizable part of the ECG was related to routine clinical practice, comprising 110,556 (28.3%) from the cardiology department and 79,256 (20.3%) from other noncardiovascular departments. Finally, the average reporting time was significantly decreased compared to reporting times without a telecardiology system (5–10 vs. 45–90 min). Conclusion: Our telecardiology system provides efficient cardiology assistance for all types, settings, and phases of cardiovascular diseases.

## Telecardiology Application for Daily Clinical Practice

Telecardiology is one of the most interesting and widespread applications of telemedicine. Allowing a real-time transmission and report of electrocardiograms (ECG), this technology represents a significant step forward in the management of time-dependent cardiovascular diseases, such as acute coronary syndromes (ACS). 

On the other hand, several guidelines and position papers recommend the use of telecardiology also for the management of chronic cardiac diseases, to ensure continuity of care on a routine basis [1]. However, in Italy, the development of telecardiology is limited by the unmet need in containing healthcare costs, inadequate reimbursement policies, and complex orography [2,3,4,5,6,7,8,9]. 

Hereafter, in this retrospective analysis, we report the results of our telecardiology system in the sanitary district of Cosenza. In particular, we report the number of ECGs transmitted in this area, categorized by year and according to the hospitals, departments (catheterization laboratories and emergency departments), and local emergency spots (first aid centers, 20 local 118-EMS stations, and 1 helicopter station) included in this program. Furthermore, we look at the effect, on a large scale, of the quality check software for improving the quality of the ECG transmitted from professional health care providers. 

**Figure 1:**
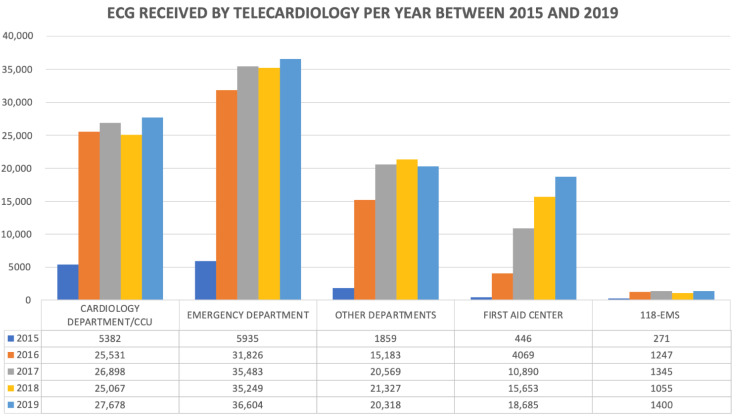
ECGs received by telecardiology per year between 2015 and 2019.

Material and Methods: The design and implementation of the telecardiology system in the sanitary district of Cosenza have been already published [10]. Briefly, the sanitary district of Cosenza is an area with a complex orography (41% mountains, 49% hills, and 10% plains), covering a region of 6700 km^2^, in the northern part of Calabria, southern Italy, with a total population of 800,000 inhabitants. This project involved a total of 8 hospitals, 9 first aid centers, 20 local 118-EMS stations, 1 helicopter station, 8 hospital emergency departments, 59 hospital departments, and 3 catheterization laboratories (Figure 1). The MUSE Cardiology Information System (General Electric, Boston, MA, USA) has been used to manage the data flow among sites, simplifying data acquirement, saving time, and operating safely with a web-based interface protected by login and password. Along with the MUSE system, the Healthcare’s Marquette 12SL ECG computerized analysis program (General Electric, Boston, MA, USA) has also been used. This system provides a fast, accurate, and validated analysis of the ECG with the measurements of heart rate, axis, intervals, and durations, according to gender and age. Furthermore, this software also indicates changes in the ECG from the previous ECG of the same patient and provides a quality check of the ECGs. All the ECGs were reviewed by cardiologists operating in the sanitary district of Cosenza accessing the MUSE Cardiology Information System with a personal credential. 

The objectives of our telecardiology system were as follows:

- a real-time connection between local 118-EMS stations, emergency departments, and coronary care units (CCU) for a fast and effective diagnosis of cardiovascular emergencies, thus limiting extra- and intra-hospital delay and referring the patient not to the nearest structure but to the most suitable one;
- to provide all structures in the area (hospitals, emergency departments, ambulances, and outpatient clinics) with electrocardiographs connected in real-time with cardiology departments, with a unique database, thus supporting clinical decision-making.

To ensure the high quality and efficient management of the telecardiology system, it was necessary to train all involved operators and to provide clinical and technical assistance 24 h, 7 days a week. To this purpose, the MUSE system was also integrated with the Marquette Hookup Advisor software (General Electric, Boston, MA, USA), a useful tool designed to detect in a real-time fashion the quality of the ECGs. This continuous feedback provided by the system was important for efficient staff training, and therefore one of the reasons underlying the fast improvement in ECG quality observed across time. This study was approved and supported by the Azienda Sanitaria Provinciale (ASP) of Cosenza, Italy. All patients provided written informed consent, which was approved by the ASP of Cosenza and complies with the laws on data protection and patient privacy. 

Results: From 1 October 2015 to 31 December 2019, a total of 389,970 ECGs were transmitted. Excluding the first warm-up period, the number of the ECGs was consistent across the time among all the units involved in the telecardiology system (Figure 1).

**Figure 2:**
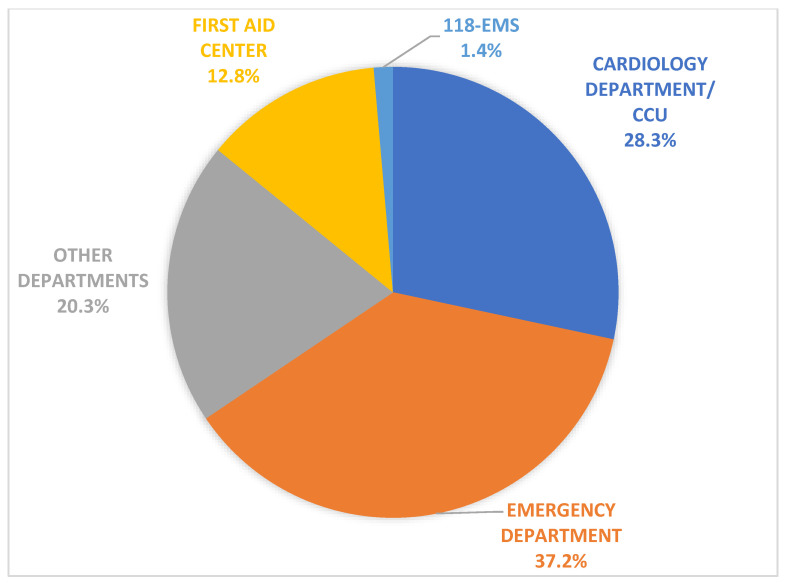
Distribution of ECGs according to department of origin.

The quality of ECGs was optimal in 52%, acceptable in 42%, and poor in 6% of the cases. For instance, the number of poor-quality ECGs was only 3% in the last 2 years, thus suggesting the importance of the ECG quality check system on continuous personnel training. Furthermore, the Marquette Hookup Advisor software has high specificity to discriminate against a “normal ECG”. We confirmed the importance of these tools in our database, of which almost 45% of all the ECGs were deemed as normal, thus supporting operators for a fast and accurate diagnosis. As depicted in Figure 2, 145,097 (37.2%) ECGs were transmitted from the emergency departments, 5318 (1.4%) from the 118-EMS stations, and 49.743 (12.8%) from the first aid centers. Of note, 1113 ECGs were diagnostic for STEMI, exhibiting a significant reduction of the time between first medical contact and access to the catheterization laboratory compared to the pre-telecardiology era. For this subset of patients, we have already shown a 21.5% time reduction for those patients coming from emergency departments and a 53.6% time reduction for those from the 118-EMS stations [11]. 

**Figure 3:**
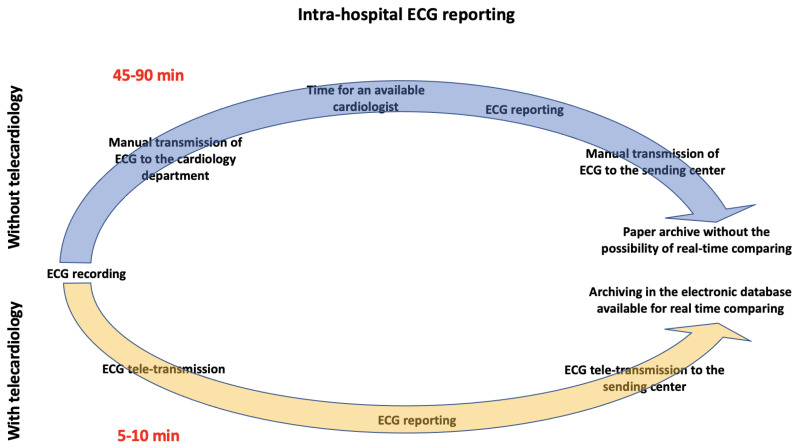
The different paths of the intra-hospital ECG with and without telecardiology.

Furthermore, a sizable proportion of the ECGs were transmitted from the cardiology 110,556 (28.3%) and other departments 79,256 (20.3%), from both outpatient clinics and hospitalized patients, to support the routine clinical consultancy and ambulatory follow-up for patients with a history of cardiovascular diseases. Finally, we estimated the time and personnel needs to manually deliver an ECG report performed in our hospital compared to the time spent using our telecardiology system. In the first case, the average reporting time was 45–90 min, while this was only 5–10 min with telecardiology. Moreover, the use of this telecardiology system also significantly reduced the amount of personnel involved compared to the conventional workflow (Figure 3).

This project is one of the largest in Europe, involving a broad territory with a complex orography, and including a heterogeneous number of structures that require diffuse informatization and continuous technical support [10].

Previous studies have shown the benefits of telecardiology in decreasing the time of intervention and improving prognosis [12,13,14,15,16]; thus, it represents a revolutionary road to providing healthcare assistance. In our sanitary district, the application of this technology for a daily clinical practice was not easy, because of infrastructural constraints and lack of cooperation from healthcare workers, who in certain cases were reluctant to leave the old for this new way to provide clinical assistance. However, defined objectives, clear timelines, and continuous technical support, this last provided in this project by General Electrics, definitely led to telecardiology becoming an essential part of our healthcare system. 

Although telecardiology is a useful tool applicable for the management of the whole spectrum of cardiovascular disease, and for emergency and non-emergency scenarios, several reports have shown that telecardiology is still primarily applied for the prehospital triage of acute myocardial infarction [17,18,19,20,21,22,23,24,25,26]. In this report, we confirm, at least in part, these observations, with almost 40% of the total ECGs being transmitted from the emergency departments or 118-EMS stations. 

Nonetheless, we observed that the majority of the transmitted ECGs underlined routine clinical activities of cardiology or other departments. In our system, ECGs recorded in every department are transmitted to a central database, where the cardiologist in charge of reporting can review and make teleconsultation. This approach allows timely and appropriate medical decisions, and minimizes the number of healthcare workers involved. This observation is of paramount importance because it supports telecardiology not only for the management of emergencies but also for a routine basis in order to achieve a more efficient clinical practice.

In aggregation, this project is a service widespread throughout the territory and hospitals, ensuring stable cardiology assistance is regularly provided for all types, settings, and phases of cardiovascular disease. 

Study limitation: This study has several limitations. First, the reason for an ECG was not available for many of the transmitted ECGs, consequently, it is not possible to report these data as well as to look, in a quantitative fashion, at the impact of telecardiology on daily clinical practice. Second, because of the retrospective nature of this study, further studies with a randomized design are warranted to confirm the impact of telecardiology in improving the ECG quality and data flow, and, even more, to evaluate its importance in supporting therapeutic strategies and costs optimization. 

## Data Availability

The data that support the findings of this study will not be made available to other researchers for purposes of reproducing the results or replicating the procedure because substudies are ongoing.

## Abbreviations

ECG	electrocardiogram
EMS	emergency medical service
ACS	acute coronary syndrome
